# Barriers to intravenous thrombolysis in acute ischemic stroke: a retrospective case report series from Honduras

**DOI:** 10.3389/fstro.2026.1946997

**Published:** 2026-10-08

**Authors:** Jesús Antonio Rodríguez Santos, Iván Pineda, Eliezer Cardona, Carlos Maradiaga, Juan Galeano, Laura Giacaman, Selvin Z. Reyes-Garcia

**Affiliations:** Faculty of Medical Sciences, National Autonomous University of Honduras (UNAH), Tegucigalpa, Honduras

**Keywords:** developing countries, health services accessibility, Honduras, stroke, thrombolytic therapy

## Abstract

**Background and purpose:**

Intravenous (IV) thrombolysis for acute ischemic stroke (AIS) is time-dependent and frequently underused in resource-limited settings. We aimed to characterize patients with AIS who did not receive IV thrombolysis at a national referral center in Honduras and to document the barriers to treatment.

**Methods:**

We conducted a retrospective, single-center, consecutive case report series of all eligible patients with AIS who were not thrombolyzed at Hospital Escuela Universitario between 1 October and 30 November 2025. We gathered demographics, socioeconomic status, admission severity, process-of-care intervals, contraindications, neuroimaging and thrombolytic availability, complications, and discharge outcomes. Contraindications were classified as absolute or relative according to the 2019 American Heart Association and American Stroke Association (AHA/ASA) guidelines. A thrombolysis eligibility cascade was the principal analysis. The study was approved by the institutional review board.

**Results:**

A total of 23 patients were included (median age 70 years; 14 [60.9%] male). Extreme poverty (≥2 unsatisfied basic needs [UBN]) was present in 14 patients (60.9%). All 23 AIS presentations screened during the period were included, and no patient received thrombolysis (institutional thrombolysis rate 0%). The median onset-to-door time was 10.0 h (IQR 7.0–15.0), and only 1 of 22 patients with a recorded time (4.5%) arrived within 4.5 h. Late or unknown onset was the most frequent absolute contraindication (21 [91.3%]); neuroimaging was available in 22 (95.7%) patients, but the thrombolytic agent was unavailable in every patient (availability 0%). In the eligibility cascade, the single patient within the window had no absolute contraindication and could not be treated because no thrombolytic agent was available. The in-hospital mortality rate was 2 (8.7%), and a good functional outcome (modified Rankin Scale [mRS] score 0–2) occurred in 5 of 20 patients with an assessable score.

**Conclusion:**

Non-administration of thrombolysis reflected late presentation and, above all, the complete unavailability of the thrombolytic agent, whereas imaging and organizational capacity were largely in place. Drug supply was the proximate, modifiable obstacle to reperfusion in this setting.

## Introduction

Stroke is the second leading cause of death and a major cause of acquired disability worldwide, and ischemic stroke accounts for the majority of cases (Feigin et al., 2021). This burden falls disproportionately on low- and middle-income countries (LMICs), where incidence is rising and case fatality remains higher than in high-income settings. Population-based data confirm a substantial and unevenly distributed burden across Latin America (Feigin et al., 2021; Delfino et al., 1997).

Intravenous (IV) thrombolysis and, in selected patients, endovascular thrombectomy are the only proven means of achieving acute reperfusion, and their benefit is steeply time-dependent (Powers et al., 2019; Lees et al., 2010). Pooled trial data show that the probability of a favorable outcome falls with every hour of delay (Lees et al., 2010), and faster in-hospital treatment is associated with lower mortality and better function (Man et al., 2020). Imaging-based selection has extended the thrombectomy window to 24 h in a minority of patients (Nogueira et al., 2018; Albers et al., 2018), yet every reperfusion strategy still depends on an uninterrupted supply of the thrombolytic agent, rapid imaging, and trained personnel. Where these are lacking, patients follow the natural history of arterial occlusion, with higher rates of disability and death (Powers et al., 2019).

Barriers to thrombolysis arise at every step of the stroke pathway. The availability of reperfusion services varies widely among countries, and many LMICs lack the systems required to deliver acute treatment (Kim et al., 2024). Prehospital delay is the most frequent reason patients fall outside the treatment window (Anees et al., 2024), and delays in door-to-imaging time reduce eligibility further (Polineni et al., 2021). Socioeconomic and health system inequities also shape who is treated, even where treatment is feasible (Denny et al., 2024). In Latin America, thrombolysis remains infrequent and heterogeneous. Single-center series from public and private hospitals report modest treatment proportions (Milpas Muñoz et al., 2025; Guevara et al., 2016), regional reviews describe persistent gaps in reperfusion delivery (Martins et al., 2023), and case fatality in stroke-unit cohorts remains high (Vences et al., 2025) even though organized stroke care and thrombectomy are achievable within regional public systems (González-Aquines et al., 2023; Martins et al., 2020). The intermittent or complete unavailability of the thrombolytic agent is a recurrent but rarely quantified barrier in resource-limited centers.

Most evidence on these barriers comes from high-income systems or from centers that already deliver reperfusion, and the majority of reports describe treated patients or compare treated with untreated patients. Patients who are never treated in low-resource referral centers have received far less attention, and the specific contribution of drug unavailability, as distinct from patient-level or temporal factors, is seldom documented. Central America, Honduras in particular, is largely absent from this literature.

We conducted a retrospective, single-center, consecutive case report series of patients with acute ischemic stroke who did not receive IV thrombolysis at a national tertiary referral hospital in Honduras. The objective was to describe their clinical and epidemiological characteristics and to document the clinical, institutional, and socioeconomic barriers to thrombolysis, summarized as a thrombolysis eligibility cascade, without inferring causation.

## Methods

### Study design and setting

We conducted an observational, descriptive, retrospective, single-center study with a consecutive case series design, reported in accordance with the Strengthening the Reporting of Observational Studies in Epidemiology (STROBE) statement (von Elm et al., 2007). The study was conducted at Hospital Escuela Universitario (HEU), the national tertiary referral center in Tegucigalpa, Honduras. The study period comprised 61 consecutive days, from 1 October to 30 November 2025. Because the design included a single group of non-thrombolyzed patients, no statistical hypothesis was tested; the within-cohort analyses described below were exploratory and hypothesis-generating.

### Participants

The source population comprised all patients evaluated for AIS in the Internal Medicine Emergency Service of HEU during the study period. Eligible patients were those with AIS confirmed by clinical assessment and neuroimaging who did not receive IV thrombolysis at HEU during the index event and whose records contained sufficient information for the primary variables. We excluded patients with hemorrhagic stroke or other non-ischemic cerebrovascular disease, those transferred to another center before neurological evaluation or the treatment decision, and those with records lacking fundamental variables. All eligible patients were enrolled consecutively (total enumeration); no probabilistic sample size calculation was performed because the study constituted a census for the period. The flow of participants from screening to the final cohort is shown in Figure 1. Screening covered all presentations to the emergency service during the study period, not only those already entered into a stroke pathway. A total of 23 AIS presentations were identified, and all were included: No patient was excluded for transfer before neurological evaluation or for insufficient records, and no patient received thrombolysis. No primary intracerebral hemorrhage was identified during the period; the only intracranial hemorrhages observed were hemorrhagic transformations of an index ischemic infarct, which are reported among the in-hospital complications. The study cohort therefore corresponds to all AIS presentations identified during the period, and the institutional thrombolysis rate for those 61 days was 0% (0 of 23, 95% CI 0–14.3). Sex was recorded as the biological sex documented in the medical record.

**Figure 1:**
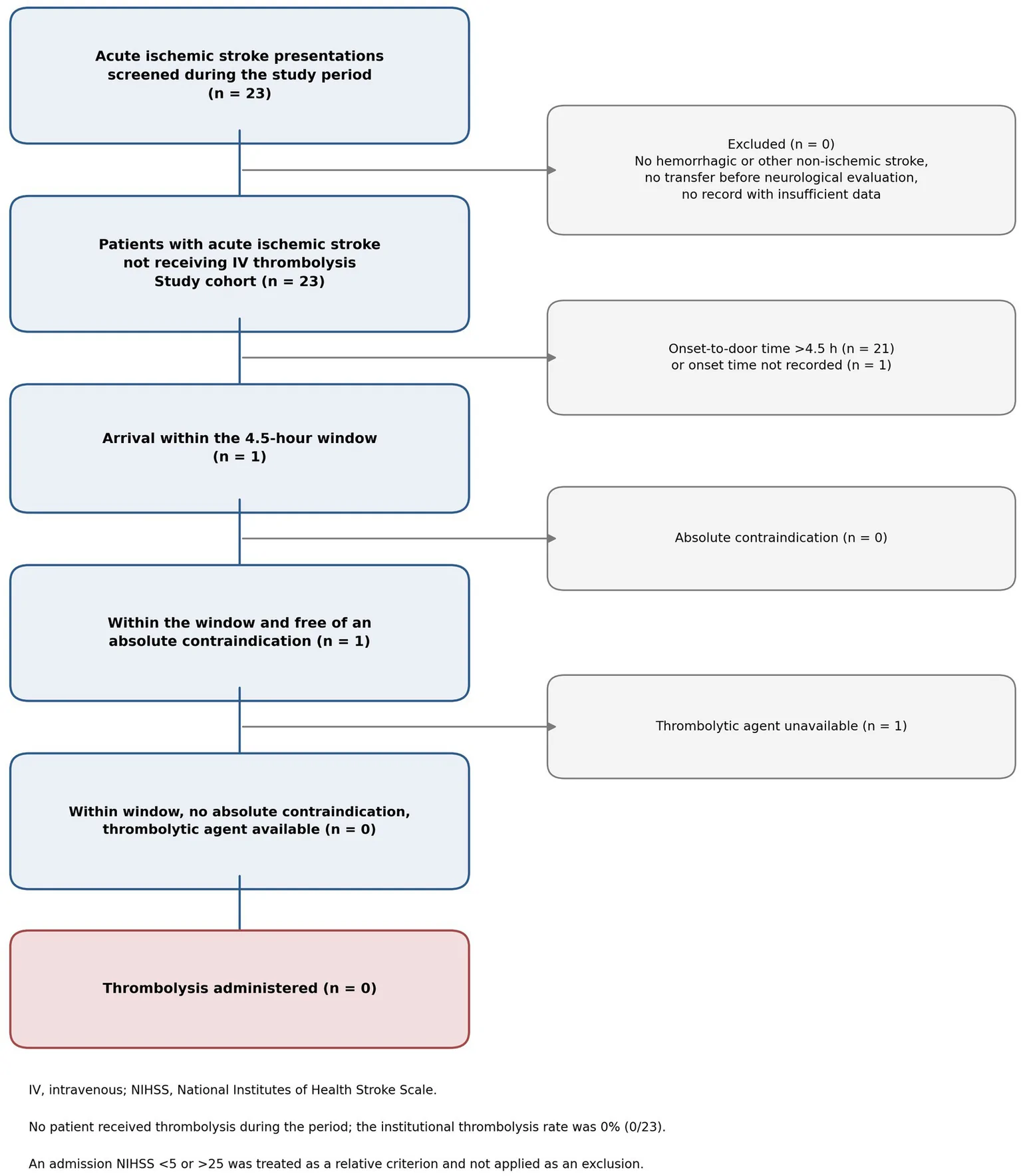
Flow of participants and thrombolysis eligibility cascade. The diagram shows screening and exclusions leading to the study cohort, followed by the sequential application of eligibility criteria: arrival within the 4.5-h window, absence of an absolute contraindication, availability of the thrombolytic agent, and thrombolysis administered. All 23 screened presentations were included, and none were excluded. Only absolute contraindications were applied at the corresponding step; an admission NIHSS below 5 or above 25 was treated as a relative criterion. One patient was within the window and free of an absolute contraindication but could not be treated because no thrombolytic agent was available. IV, intravenous; NIHSS, National Institutes of Health Stroke Scale.

### Variables and data sources

Data were extracted from clinical records, emergency admission sheets, the thrombolysis registry, and departmental databases using a structured form and were complemented by a structured interview with the patient’s accompanying relative to reconstruct the UBN index. Sociodemographic variables were age, sex, department of residence, and socioeconomic status (UBN). Admission severity was recorded using the National Institutes of Health Stroke Scale (NIHSS) and, due to the data collection instrument, was captured in three ordinal categories (<5, 5–25, >25) rather than as a continuous score. Process-of-care intervals were recorded as three independent variables and converted to hours: onset-to-door (symptom onset to emergency department registration), door-to-imaging (registration to acquisition of neuroimaging), and door-to-neurological evaluation (registration to the first assessment by the neurology service). Door-to-imaging were and door-to-neurological evaluation extracted from separate source entries and were not derived from one another; because neurological assessment and imaging are requested in parallel at our center, either may precede the other in an individual patient.

Barriers comprised contraindications to thrombolysis, availability of neuroimaging, availability of the thrombolytic agent (recombinant tissue plasminogen activator), and installed capacity (existence of an institutional stroke protocol and trained personnel). Contraindications were classified according to the 2019 AHA/ASA guidelines (Powers et al., 2019) and, in this revision, were separated into absolute criteria (symptom onset beyond 4.5 h or of unknown time, extensive stroke within the preceding 3 months, and blood pressure refractory to treatment above 185/110 mmHg) and relative criteria requiring individual clinical judgment. In line with the guidelines, an admission NIHSS score below 5 or above 25 was treated as a relative criterion rather than an absolute exclusion, since the decision in patients with low scores depends on whether the deficit is disabling and the evidence in very severe strokes reflects an unfavorable risk–benefit balance rather than a formal prohibition. Only absolute criteria were applied in the eligibility cascade. Cardiovascular risk factors, in-hospital complications, vital status at discharge, NIHSS score at 24–72 h, discharge NIHSS score, and the mRS score at discharge (Banks and Marotta, 2007) were also recorded.

### Data management

All direct identifiers were removed before analysis, and each record was assigned an anonymized code. Free-text time fields were parsed to hours using a predefined convention; ambiguous or absent entries were treated as missing. Missing data were handled using complete-case analysis, and the analyzed denominator was reported for every variable. A documented data correction registry was maintained: One discharge NIHSS score value recorded as 38 in a surviving patient was confirmed by the investigators to be a data entry error and corrected to 18; discharge NIHSS score was set to missing for the two patients who died, because the score is not valid at death; and, for one patient whose onset-to-door time had been entered as “4 h,” the investigators confirmed on chart review that symptom onset had occurred 5 h before arrival, so the patient was classified as outside the 4.5-h window. The median and interquartile range (IQR) of the onset-to-door time remained unchanged by this correction. The complete data cleaning and analysis pipeline is provided as a reproducible supplement.

### Statistical analysis

Analyses were descriptive and prioritized estimation with confidence intervals over significance testing. All categorical variables reported in Tables 1–3 were summarized as absolute and relative frequencies (n, %) with 95% Wilson confidence intervals (CIs), which are appropriate for small samples (Wilson, 1927); intervals are therefore presented for every proportion rather than for a selected subset. Continuous time variables, given their marked right skew, were summarized as median, IQR, and range; ordinal variables (admission NIHSS category and mRS score) were summarized as frequencies and, where applicable, median (IQR). The principal analysis was a thrombolysis eligibility cascade quantifying the sequential loss of potentially treatable patients (total, then arrival within 4.5 h, then absence of an absolute contraindication, then availability of the thrombolytic agent, then treatment). Prespecified within-cohort bivariate relationships were explored only as hypothesis-generating analyses, with the effect size and its 95% CI as the primary result and the *p*-value reported as secondary and non-interpretive. We used the Spearman rank correlation (rho; CI by the Fisher z transformation). Tests of factors associated with non-thrombolysis, multivariable modeling, survival analysis, and causal inference were considered not feasible given the single-group design, the constant outcome, and the small sample. Analyses were performed using Python 3.12 (pandas, numpy, scipy, statsmodels, and pingouin).

**Table 1:** Baseline characteristics of patients with acute ischemic stroke not treated with intravenous thrombolysis (*n* = 23).

Characteristic	n (%) [95% CI]
Age, years—median (IQR)	70 (62.5–78.0)
Male sex	14 (60.9) [40.8–77.8]
Socioeconomic status (UBN)	
No poverty (0 UBN)	4 (17.4) [7.0–37.1]
Poverty (1 UBN)	4 (17.4) [7.0–37.1]
Extreme poverty (≥2 UBN)	14 (60.9) [40.8–77.8]
Not available	1 (4.3) [0.8–21.0]
Admission NIHSS score category	
<5	2 (8.7) [2.4–26.8]
5–25	19 (82.6) [62.9–93.0]
>25	2 (8.7) [2.4–26.8]
Cardiovascular risk factors	
Hypertension	22 (95.7) [79.0–99.2]
Diabetes mellitus	8 (34.8) [18.8–55.1]
Established cardiovascular disease	4 (17.4) [7.0–37.1]
Smoking	1 (4.3) [0.8–21.0]
Anticoagulant use	1 (4.3) [0.8–21.0]
Dyslipidemia	0 (0) [0.0–14.3]
None	1 (4.3) [0.8–21.0]

CI, confidence interval; IQR, interquartile range; NIHSS, National Institutes of Health Stroke Scale; UBN, unsatisfied basic needs. Confidence intervals are Wilson intervals for proportions. The admission NIHSS score was recorded categorically using the data collection instrument.

**Table 2:** Process-of-care intervals and barriers to intravenous thrombolysis (*n* = 23).

Variable	n (%) [95% CI] or median (IQR)
Onset-to-door time, h—median (IQR; range)	10.0 (7.0–15.0; 4.0–96.0)
Arrival within 4.5 h (*n* = 22 with recorded time)	1 (4.5) [0.8–21.8]
Door-to-imaging time, h—median (IQR); *n* = 21	3.0 (2.0–5.0)
Door-to-neurology time, h—median (IQR; range); *n* = 21	3.0 (1.0–6.0; 0.3–120.0)
Any contraindication (absolute or relative)	23 (100) [85.7–100]
Absolute contraindications	
Onset >4.5 h or of unknown time	21 (91.3) [73.2–97.6]
Extensive stroke within 3 months	1 (4.3) [0.8–21.0]
Blood pressure refractory to treatment >185/110 mmHg	1 (4.3) [0.8–21.0]
Relative criteria	
NIHSS score at admission <5 or >25	4 (17.4) [7.0–37.1]
Neuroimaging available	22 (95.7) [79.0–99.2]
Thrombolytic agent available	0 (0) [0.0–14.3]
Institutional protocol and trained staff	22 (95.7) [79.0–99.2]

CI, confidence interval; IQR, interquartile range; NIHSS, National Institutes of Health Stroke Scale. Confidence intervals are Wilson intervals for proportions; denominators are stated where they differ from 23. Contraindication categories are not mutually exclusive. Contraindications were classified according to the 2019 AHA/ASA guidelines. An admission NIHSS score of <5 or >25 is reported as a relative criterion requiring individual clinical judgment and was not applied as an exclusion in the eligibility cascade.

**Table 3:** In-hospital complications and discharge outcomes (*n* = 23).

Outcome	n (%) [95% CI] or median (IQR)
In-hospital complications	
None	11 (47.8) [29.2–67.0]
Intracranial hemorrhage	4 (17.4) [7.0–37.1]
Aspiration pneumonia	3 (13.0) [4.5–32.1]
Cerebral edema	2 (8.7) [2.4–26.8]
Other	3 (13.0) [4.5–32.1]
Vital status at discharge	
Alive	21 (91.3) [73.2–97.6]
Dead	2 (8.7) [2.4–26.8]
NIHSS score at 24–72 h, survivors—median (IQR)	11 (8–21)
NIHSS score at discharge, survivors—median (IQR)	11 (8–18)
mRS score at discharge	
0–2 (good)	5 (21.7) [9.7–41.9]
3–5 (poor)	13 (56.5) [36.8–74.4]
6 (death)	2 (8.7) [2.4–26.8]
Not assessable	3 (13.0) [4.5–32.1]

CI, confidence interval; IQR, interquartile range; mRS, modified Rankin Scale; NIHSS, National Institutes of Health Stroke Scale. Confidence intervals are Wilson intervals for proportions. Proportions for the mRS are calculated on the whole cohort (*n* = 23); Figure 2 shows the distribution among the 20 patients with an assessable score. “Other” complications included dysphagia, multi-infarct disease, and one non-standard descriptor. NIHSS score at discharge was set to missing for the two patients who died.

### Ethics

The study was approved by the institutional research ethics committee and conducted in accordance with the principles of the Declaration of Helsinki. Because it was a retrospective record-based study, a waiver of individual informed consent was granted. Confidentiality was preserved through alphanumeric coding and anonymized data handling.

## Results

### Cohort and baseline characteristics

A total of 23 patients with AIS who did not receive IV thrombolysis were included (Figure 1). The median age was 70 years (IQR 62.5–78.0; range 46–89), and 14 patients (60.9%; 95% CI 40.8–77.8) were male. The majority of patients resided in the Francisco Morazán Department, the capital of the country and the hospital’s primary catchment area. Extreme poverty (≥2 unsatisfied basic needs) was present in 14 patients (60.9%; 95% CI 40.8–77.8). Cardiovascular risk factors were highly prevalent: hypertension in 22 patients (95.7%; 95% CI 79.0–99.2) and diabetes mellitus in 8 patients (34.8%; 95% CI 18.8–55.1), with a median of two risk factors per patient (range 0–3). An admission NIHSS score category of 5–25 was recorded in 19 patients (82.6%; 95% CI 62.9–93.0), with 2 patients (8.7%) in each of the <5 and >25 categories. Both patients in the <5 category had a recorded NIHSS score of 3, comprising mild hemiparesis (2 points) and mild aphasia (1 point). Baseline characteristics are shown in Table 1.

### Process-of-care intervals and barriers

The median onset-to-door time was 10.0 h (IQR 7.0–15.0; range 4.0–96.0), and only 1 of 22 patients with a recorded time (4.5%; 95% CI 0.8–21.8) arrived within the 4.5-h window. The median door-to-imaging time was 3.0 h (IQR 2.0–5.0; *n* = 21), and the median door-to-neurological-evaluation time was also 3.0 h (IQR 1.0–6.0; range 0.3–120.0; *n* = 21). The two intervals were recorded independently, and their distributions differed, with neurological evaluation preceding imaging in 11 of the 19 patients in whom both were available and following it in four patients; identical values occurred in four patients.

At least one contraindication, absolute or relative, was documented in every patient (23 patients; 100%; 95% CI 85.7–100). The most frequent absolute criterion was symptom onset beyond 4.5 h or of unknown time (21 patients; 91.3%; 95% CI 73.2–97.6); extensive stroke within 3 months and refractory hypertension were each recorded once (4.3%; 95% CI 0.8–21.0). An admission NIHSS score below 5 or above 25, classified here as a relative criterion, was present in four patients (17.4%; 95% CI 7.0–37.1). Neuroimaging was available in 22 patients (95.7%; 95% CI 79.0–99.2), and an institutional protocol with trained personnel was present in 22 patients (95.7%; 95% CI 79.0–99.2). The thrombolytic agent, however, was unavailable in every patient (availability 0%; 95% CI 0–14.3). Process intervals and barriers are detailed in Table 2.

In the thrombolysis eligibility cascade (Figure 1), the cohort of 23 decreased to 1 patient who arrived within the 4.5-h window. This patient had no absolute contraindication and could not be treated because no thrombolytic agent was available, so the number of patients treated was 0.

### Complications and discharge outcomes

In-hospital complications occurred in 12 patients (52.2%; 95% CI 33.0–70.8); the most frequent were intracranial hemorrhage (four patients; 17.4%; 95% CI 7.0–37.1), aspiration pneumonia (three patients; 13.0%; 95% CI 4.5–32.1), and cerebral edema (two patients; 8.7%; 95% CI 2.4–26.8). All four intracranial hemorrhages were hemorrhagic transformations of the index infarct, confirmed by non-contrast computed tomography; magnetic resonance imaging was not available during the study period. They were detected on days 4, 5, 7, and 8 of hospitalization, and all four patients were symptomatic, each accompanied by neurological deterioration attributable to the hemorrhage. The NIHSS scores recorded after the event were 10, 12, 34, and 35; the patient scoring 35 died, and the other three patients were discharged alive with residual disability. The in-hospital mortality rate was 2 (8.7%; 95% CI 2.4–26.8). Among the 21 survivors, the median NIHSS score at 24–72 h was 11 (IQR 8–21), and the median discharge NIHSS score was 11 (IQR 8–18). A good functional outcome (mRS 0–2) was recorded in five patients, corresponding to 21.7% of the whole cohort (95% CI 9.7–41.9) and to 5 of the 20 patients with an assessable mRS score (Figure 2); the mRS score was not assessable in 3 patients (13.0%; 95% CI 4.5–32.1). Complications and outcomes are presented in Table 3.

**Figure 2:**
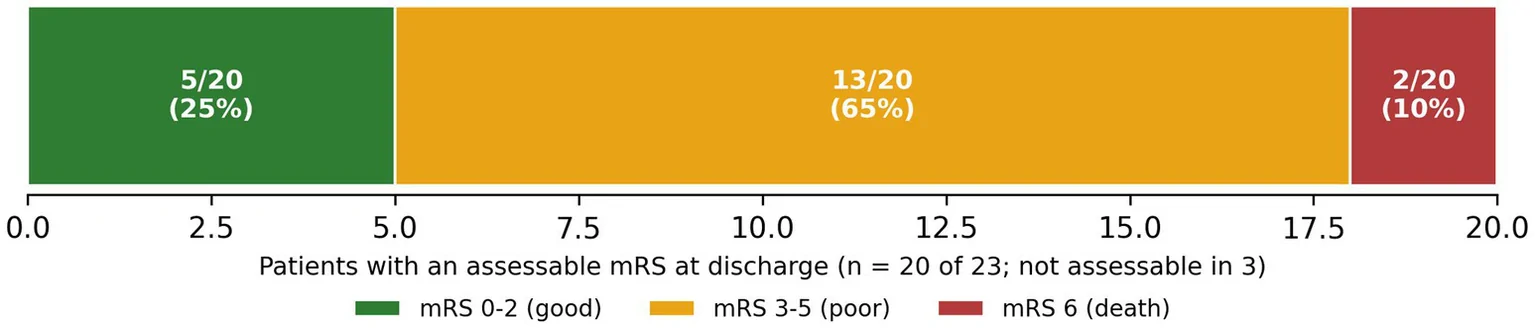
Distribution of the modified Rankin Scale (mRS) at discharge among 20 of 23 patients with an assessable score (mRS not assessable in three patients): good outcome (mRS 0–2, *n* = 5), poor outcome (mRS 3–5, *n* = 13), and death (mRS 6, *n* = 2). Percentages are calculated on the 20 patients with an assessable score; Table 3 reports the same counts as proportions of the whole cohort of 23. mRS, modified Rankin Scale.

### Exploratory analyses

In prespecified, hypothesis-generating analyses, greater early severity was associated with worse functional outcome: NIHSS score at 24–72 h correlated with discharge mRS score (Spearman rho 0.79; 95% CI 0.53–0.92), as did the admission NIHSS score category (rho 0.46; 95% CI 0.02–0.75). The corresponding *p*-values were secondary and are not used to interpret these results.

## Discussion

### Principal findings

This consecutive series of 23 patients with acute ischemic stroke who were not thrombolyzed is dominated by two findings. Patients presented late: The median onset-to-door time was 10 h, and only one patient reached the hospital within the 4.5-h window. In addition, the thrombolytic agent was unavailable for every patient in the cohort, and no patient was thrombolyzed during the 61-day period. The eligibility cascade shows that these barriers were not redundant: The single patient who arrived within the window had no absolute contraindication and remained untreated solely because no drug was available. Neuroimaging and an institutional stroke protocol with trained staff were available in almost all patients, which places the absence of the drug, rather than diagnostic or organizational capacity, as the proximate system-level obstacle. The cohort had a high prevalence of extreme poverty and vascular risk factors, an admission NIHSS score category of 5–25 in the majority of patients, and an in-hospital mortality rate of 8.7%.

### Comparison with previous literature

Because every AIS presentation screened during the period entered the cohort and none was treated, the institutional thrombolysis rate for those 61 days was 0% (0 of 23; 95% CI 0–14.3), and pharmacological reperfusion was effectively unavailable. This stands in contrast to regional reports in which the drug was available and a measurable, if modest, proportion of patients were treated, such as single-center series from Mexico and Chile (Milpas Muñoz et al., 2025; Guevara et al., 2016). The pattern is consistent with global analyses showing that the availability of reperfusion services, and not clinical eligibility alone, determines whether patients can be treated (Kim et al., 2024), and it gives a concrete, patient-level illustration of the supply-side gap described in regional reviews (Martins et al., 2023).

The median onset-to-door time of 10 h is considerably longer than the delays reported in other resource-limited tertiary centers (Anees et al., 2024), and the proportion arriving within the thrombolysis window (9%) is correspondingly low. Independent of drug supply, prehospital delay alone would therefore have excluded the majority of these patients, consistent with the established time dependence of reperfusion benefit (Lees et al., 2010). Neuroimaging, by contrast, was almost universally available; the median door-to-imaging time of 3 h nonetheless exceeds guideline targets and the times reported in organized systems (Polineni et al., 2021). Imaging access was not the limiting step in this setting, which distinguishes the local bottleneck from the imaging-related disparities described elsewhere (Polineni et al., 2021).

The in-hospital mortality rate (8.7%) is within the range reported for stroke-unit cohorts in Latin America (Vences et al., 2025), and the correlation between early neurological severity and discharge disability is consistent with the recognized prognostic value of the NIHSS (Farooque et al., 2020). This relationship was exploratory and hypothesis-generating rather than confirmatory.

### Clinical and health system implications

One implication follows directly from the data. In a center where imaging and trained personnel are already in place, securing an uninterrupted supply of the thrombolytic agent is the single most consequential intervention; without it, investment in pathways and training cannot translate into treatment. Reducing prehospital delay through public awareness and emergency prenotification would enlarge the pool of window-eligible patients, but only once the drug is available (Kim et al., 2024; Denny et al., 2024). The concentration of extreme poverty in the cohort places these system failures within broader inequities in stroke care (Denny et al., 2024). Quality-improvement strategies have improved adherence to acute stroke therapies in Latin American hospitals (Machline-Carrion et al., 2019), which suggests that organizational gains are achievable once the structural prerequisite of drug availability is met.

### Interpretation of the eligibility cascade

The eligibility cascade makes the sequence of attrition explicit. Of the 23 patients, the pool fell to one patient after the time window was applied, and this patient had no absolute contraindication yet could not be treated because the agent was unavailable. Restricting the cascade to absolute criteria is important here: The single window-eligible patient had an admission NIHSS score above 25, so counting a very high score as an exclusion would have closed the cascade one step earlier and concealed the fact that an otherwise eligible patient was denied treatment by drug supply alone. The same consideration applies to the two patients with an NIHSS score of 3, whose deficits included aphasia and would therefore not have justified withholding treatment on severity grounds if they had presented within the window. Identifying where patients are lost, first to prehospital delay and then to an absolute supply barrier, defines the rate-limiting step for stroke systems planning in this setting. The cascade describes the cohort and does not estimate the effect of any single factor on the probability of treatment.

### Strengths

The design has corresponding strengths. Consecutive patients were included over a defined period (total enumeration), which limits selection within the non-thrombolyzed group, and the data reflect routine practice. The outcomes were measured using standardized instruments (the NIHSS and the modified Rankin Scale). Barriers were documented explicitly and were summarized in an eligibility cascade rather than inferred. Analyses prioritized estimation with confidence intervals over significance testing, missing data were reported in full, and the deidentified dataset and analysis code are available for reproducibility.

### Limitations

The study has important limitations. The design was retrospective and single-center, and the cohort was small (*n* = 23) and confined to a 61-day period that may have coincided with a specific interval of drug shortage, limiting external validity. Because no patient was treated during the period, there is no comparator group of treated patients, and no patient-level factor is claimed to be associated with non-treatment, since the outcome was constant by design. Information bias is possible: data were extracted from records, symptom-onset times were frequently self-reported and at times recorded coarsely, the admission NIHSS score was captured categorically rather than as a continuous score, and the socioeconomic index was reconstructed from an informant interview. The categorical NIHSS score limited the adjudication of disabling versus non-disabling deficits, although chart review established that both patients with a score below 5 had aphasia in addition to mild hemiparesis. We treated the NIHSS score criterion as relative and excluded it from the cascade. Hemorrhagic transformations were confirmed by computed tomography alone, since magnetic resonance imaging was not operational, which may have limited the detection of small or early hemorrhages. Missing data affected several time and outcome variables and were handled by complete-case analysis. The bivariate analyses were exploratory, without correction for multiplicity or causal interpretation. The study is therefore best read as a descriptive characterization of barriers in one setting rather than an estimate of their effects.

### Future directions

Prospective, multicenter stroke registries that capture all acute ischemic strokes, treated and untreated, would allow true thrombolysis rates to be calculated and barriers to be analyzed more robustly. Routine monitoring of thrombolytic supply, combined with quality-improvement initiatives of the kind already shown to work in Latin America (Machline-Carrion et al., 2019), could address the structural gap identified here. Regional systems analyses, in the longer term, the evaluation of access to thrombectomy (González-Aquines et al., 2023; Martins et al., 2020), would help define a realistic reperfusion strategy for resource-limited Central American centers.

## Conclusion

In this single-center, consecutive case report series with acute ischemic stroke who did not receive intravenous thrombolysis, the predominant documented barrier was the complete unavailability of the thrombolytic agent, compounded by late presentation that left only a small minority within the treatment window, while imaging and organizational capacity were largely in place. These descriptive findings identify drug supply as the proximate and modifiable obstacle to reperfusion in this setting. They should be read as hypothesis-generating for system-level evaluation rather than as evidence of causal association.

## Data Availability

The raw data supporting the conclusions of this article will be made available by the authors, without undue reservation.
